# *Fas/CD95* Deficiency in *Apc^Min/+^* Mice Increases Intestinal Tumor Burden

**DOI:** 10.1371/journal.pone.0009070

**Published:** 2010-02-05

**Authors:** Hector Guillen-Ahlers, Mark A. Suckow, Francis J. Castellino, Victoria A. Ploplis

**Affiliations:** 1 W. M. Keck Center for Transgene Research, University of Notre Dame, Notre Dame, Indiana, United States of America; 2 Department of Chemistry and Biochemistry, University of Notre Dame, Notre Dame, Indiana, United States of America; 3 Walther Cancer Research Center, University of Notre Dame, Notre Dame, Indiana, United States of America; 4 Freimann Life Science Center, University of Notre Dame, Notre Dame, Indiana, United States of America; Duke-NUS Graduate Medical School, Singapore

## Abstract

**Background:**

Fas, a member of the tumor necrosis family, is responsible for initiating the apoptotic pathway when bound to its ligand, Fas-L. Defects in the Fas-mediated apoptotic pathway have been reported in colorectal cancer.

**Methodology/Principal Findings:**

In the present study, a variant of the *Apc^Min/+^* mouse, a model for the human condition, Familial Adenomatous Polyposis (FAP), was generated with an additional deficiency of Fas *(Apc^Min/+^/Fas^lpr^)* by cross-breeding *Apc^Min/+^* mice with Fas deficient (*Fas^lpr^*) mice. One of the main limitations of the *Apc^Min/+^* mouse model is that it only develops benign polyps. However, *Apc^Min/+^/Fas^lpr^* mice presented with a dramatic increase in tumor burden relative to *Apc^Min/+^* mice and invasive lesions at advanced ages. Proliferation and apoptosis markers revealed an increase in cellular proliferation, but negligible changes in apoptosis, while p53 increased at early ages. Fas-L was lower in *Apc^Min/+^*/*Fas^lpr^* mice relative to *Apc^Min/+^* cohorts, which resulted in enhanced inflammation.

**Conclusions/Significance:**

This study demonstrated that imposition of a Fas deletion in an *Apc^Min/+^* background results in a more aggressive phenotype of the *Apc^Min/+^* mouse model, with more rapid development of invasive intestinal tumors and a decrease in Fas-L levels.

## Introduction

Apoptosis is a regulated process that eliminates individual cells that are damaged or infected. There are several signals capable of triggering apoptosis, of which the activation of Fas/CD95/Apo-1 receptor (Fas) by Fas ligand (Fas-L) is the most studied [1]. Most information about the Fas pathway is based on the immune system, where Fas is highly expressed in activated T and B cells, thymocytes, and lung and liver cells [2]. Cloning of the Fas receptor (*lpr*) [3] and the Fas-L (*gld*) [4] genes led to studies involving their potential roles in the activation of the apoptotic pathway by death receptors. From these studies, a potential mechanism evolved in which interaction of Fas-L with Fas results in a conformational change of the receptor resulting in the assembly of the death-inducing signaling complex, which is able to recruit and cleave procaspase-8 (reviewed in [5]).

During colon cancer, regulation of the Fas system facilitates tumor development. Some studies have shown that resistance of colon carcinoma cells to apoptosis can be attributed to the elevated expression of Fas-associated phosphatase-1 [6], which inhibits Fas signaling by binding to the cytoplasmic tail of Fas. Another factor that may contribute to apoptosis resistance is incomplete Fas surface expression, with appropriate Fas mRNA levels but deficient posttranslational processing, attenuating its cell surface expression and remaining inactivated [7]. Amplification of a decoy receptor for Fas-L in lung and colon cancer has also been reported [8]. A number of investigations have proposed a “Fas counterattack”, which is thought to be an anti-host tumor-derived response [9], where lymphocyte proliferation is compromised by tumor cells expressing Fas-L. This induces apoptosis in the lymphocytes, sparing tumor cells due to another mechanism, i.e., low Fas surface expression [10], [11], [12]. This notion however, remains controversial, and has been refuted by some investigators [13] and contradicted by other *in vivo* studies [14], [15], [16], [17].

The adenomatous polyposis coli multiple intestinal neoplasia (*Apc^Min/+^*) mouse model presents phenotypes reminiscent of Familial Adenomatous Polyposis (FAP) in humans [18], [19]. Patients with FAP develop multiple adenomas in the large intestine, which lead to the development of malignant adenocarcinomas. The relevance of the *Apc^Min/+^* mouse model is that most colorectal cancers also show alterations in expression of the *Apc* gene [20], [21]. Apc is a gatekeeper that regulates the levels of β-catenin [22], a transcription factor that has *Matrix metalloproteinase-7* [23] among its target genes. In the current study, *Apc^Min/+^* mice were crossed with Fas deficient (*Fas^lpr^*) mice to generate *Apc^Min/+^*/*Fas^lpr^* mice in order to study the effect of a disrupted Fas-mediated apoptotic machinery on tumor development and progression. The results are summarized herein.

## Material and Methods

### Mice and Tissue Processing

The animal protocols used in this study were approved by the University of Notre Dame Institutional Animal Care and Use Committee. Male *Apc^Min/+^* and Fas-deficient (*Fas^lpr^*) mice (5 weeks old) were purchased from Jackson Laboratories (Bar Harbor, ME). All animals were fed a rodent chow diet. For these studies, *Apc^Min/+^* mice were crossed with *Fas^lpr^* mice to generate *Apc^Min/+^/Fas^lpr/+^* mice, which were then bred to generate *Apc^Min/+^/Fas^lpr^* mice. Male *Apc^Min/+^* and *Apc^Min/+^/Fas^lpr^* mice (8, 12, 16, 20, and 30 weeks) in a C57BL/6 background, were used for all analyses. Tumor counting was performed under a dissecting microscope by investigators blinded to the genotype. Intestines were opened longitudinally, cleaned, Swiss-rolled, fixed with periodate-lysine-paraformaldehyde (PLP), and embedded in paraffin.

### Blood Analysis

Blood extracted intravenously from individual mice was treated with EDTA and an aliquot (50 µl) was applied to an automated CBC analyzer (Hemavet HV950FS, Drew Scientific, Oxford, CT, USA) in order to determine the number of leukocytes (lymphocytes, neutrophils, monocytes), erythrocytes (red blood cells, hemoglobin and hematocrit), and thrombocytes (platelets).

### Histochemistry and Immunohistochemistry

Serial sections of paraffin-embedded tissue were cut for haematoxylin and eosin (H&E) staining and for immunostaining. Active caspase-3 and Fas-L were identified utilizing polyclonal rabbit-anti-human active caspase-3 and Fas-L antibodies (Abcam, Cambridge, MA), followed by HRP-conjugated goat-anti-rabbit IgG (Santa Cruz Biotechnology, Santa Cruz, CA). PCNA was identified utilizing a monoclonal mouse-anti-human PCNA antibody (BioGenex, San Ramon, CA) as the primary antibody, followed by HRP-conjugated rabbit-anti-mouse IgG (Santa Cruz Biotechnology). A mouse monoclonal-anti-mouse p53 antibody (Abcam) was used to detect p53, followed by HRP-conjugated rabbit-anti-mouse IgG (AbD Serotec, Oxford, UK). Total Akt and phosphorylated Akt were identified utilizing polyclonal rabbit-anti-mouse Akt and pAkt antibodies (Abcam and Cell Signaling, Danvers, MA, respectively), followed by biotinylated swine-anti-rabbit IgG (Dako, Carpinteria, CA) and HRP-conjugated streptavidin (BioGenex). CD45 and Mac-3 were identified utilizing a monoclonal rat-anti-mouse antibodies for CD45 and Mac-3 (Pharmingen, San Diego, CA), followed by a biotinylated rabbit-anti-mouse IgG (Dako, Carpinteria, CA) and HRP conjugated streptavidin (Jackson, West Grove, PA). Phosphorylated Foxo3a was identified utilizing a polyclonal rabbit-anti-rat Foxo3a (phosphor S253) antibody (Abcam), followed by HRP-conjugated goat-anti-rabbit IgG (Santa Cruz Biotechnology). All stains were then developed in 3, 3′-diaminobenzidine (DAB) and a haematoxylin QS counterstain was applied (Vector Laboratories, Burlington, CA).

### Stain Quantification and Statistical Analysis

Immunohistochemical stains were quantified using Spectrum Plus (Version 9.0.748.1518) and ImageScope software (Aperio Technologies, Vista, CA). Slides were scanned and loaded into an electronic database at 20X by ScanScope CS (Aperio Technologies). For each stain, algorithms were developed to analyze either the percent positive stained area per tumor area (Fas-L, Akt and pAkt) or the number of positive cells per tumor area (PCNA, caspase-3, p53, CD45, Mac-3 and p-Foxo3a).

Scanned slides were viewed in ImageScope where regions were drawn around each tumor and analyzed with Spectrum software, using algorithms designed for each stain. Stains using percent positive area were analyzed using color deconvolution algorithms. Stains requiring the number of positive cells per tumor area were analyzed using algorithms that separate color by optical density and also separate cells by outer membrane size and roundness. Cells that fit within the parameters set for desired cell size and shape and stain were counted, as was the area of each tumor region. The average number of cells per tumor area was then determined.

### Statistical Analysis

For survival studies, the data were analyzed using the Kaplan-Meier treatment and the comparison of survival between both genotypes was performed using the log-rank test with Prism 4 software (GraphPad Software, La Jolla, CA). The Student's t-test was used for comparison of single pairs.

## Results

### Fas Deletion in *Apc^Min/+^* Mice Increases the Size and Number of Intestinal Adenomas

**Figure 1:**
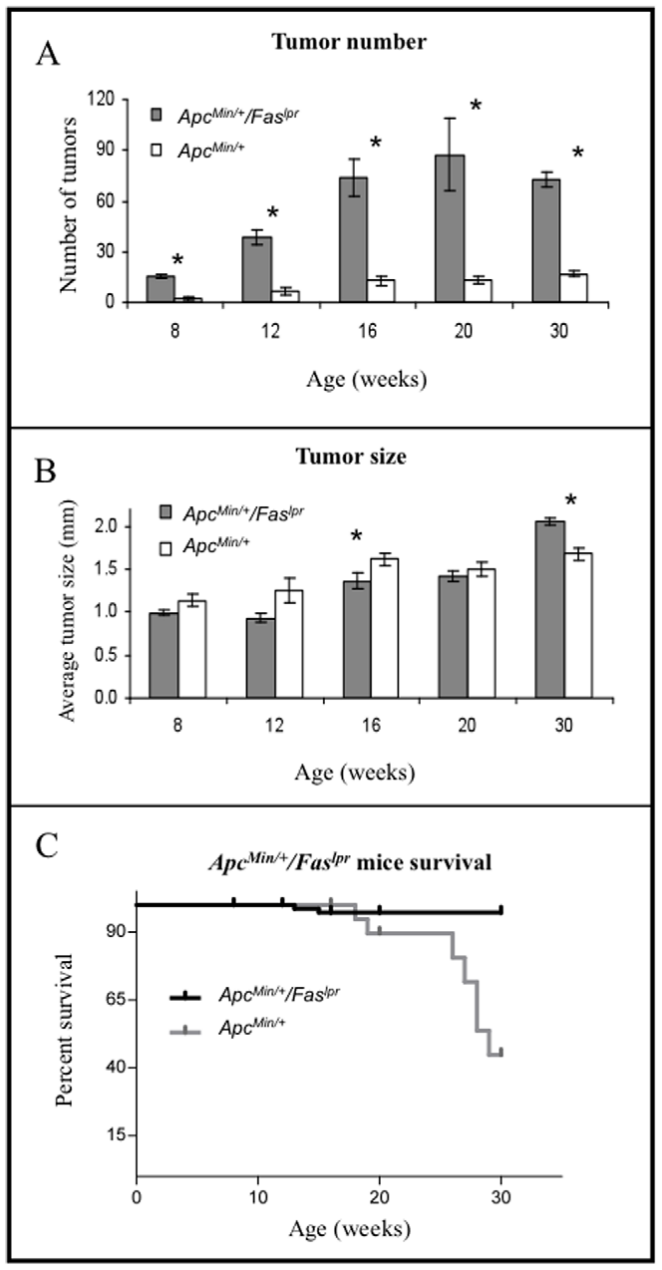
Tumor burden and survival. (**A**) Intestinal tumor number, (**B**) tumor size, and (**C**) survival rate of *Apc^Min/+^* and *Apc^Min/+^/Fas^lpr^* mice. Error bars in panels (**A**) and (**B**) represent standard error of the mean.

**Figure 2:**
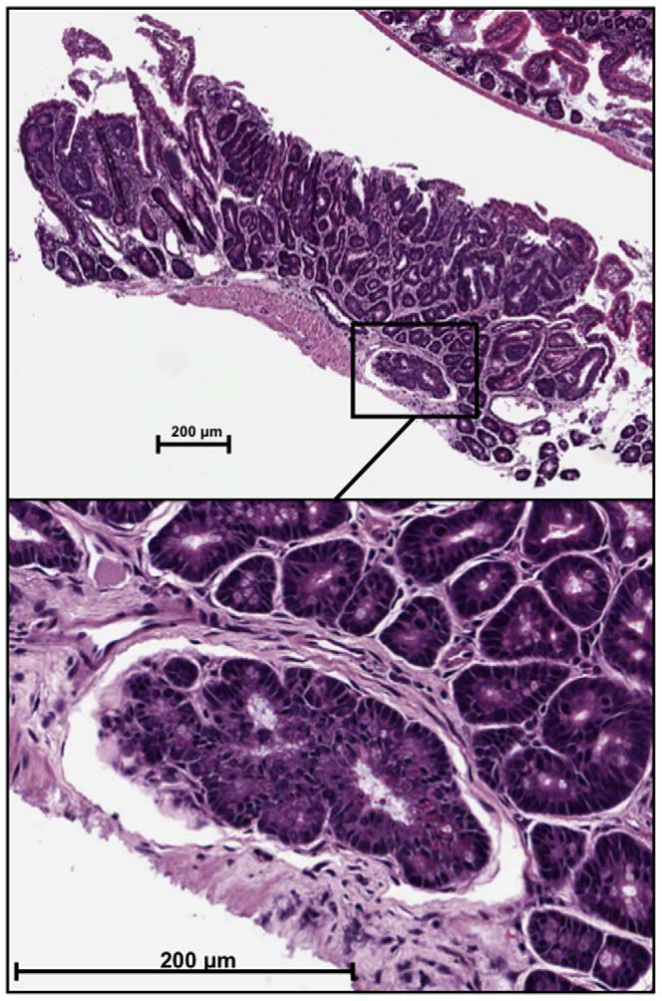
Invasive lesions. H&E stains of intestinal adenoma sections of *Apc^Min/+^/Fas^lpr^* mice at 30 weeks. The panel on the top represents an entire polyp with evidence of an invasive lesion, further magnified in the lower panel. Invasive lesions were analyzed by a veterinary pathologist and were not identified as areas of herniation, which is common in *Apc^Min/+^* mice.

Based on preliminary transcriptional profiling results of human colon cancer samples, where a strong downregulation of a FAS-related receptor was observed (data not shown), along with published reports of compromised Fas-mediated apoptosis in colon cancer, *Apc^Min/+^/Fas^lpr^* mice were generated to determine the effects of a Fas deficiency in the *Apc^Min/+^* mouse model. Intestines harvested at various timepoints revealed that *Apc^Min/+^/Fas^lpr^* mice developed dramatically more intestinal adenomas than *Apc^Min/+^*mice, increasing rapidly with time (p<0.00001 at 8 weeks, p = 0.0072 at 12 weeks, p = 0.0012 at 16 weeks, p = 0.00019 at 20 weeks and p<0.00001 at 30 wks, n = 4–10 for *Apc^Min/+^/Fas^lpr^* and 5–21 for *Apc^Min/+^/Fas^lpr^*), and reaching a plateau of slightly more than 70 adenomas after 16 weeks of age (Fig. 1A). The largest difference was observed at 20 weeks, where *Apc^Min/+^/Fas^lpr^* mice had on average 87 intestinal adenomas per mouse compared to 13 in the *Apc^Min/+^* mice. The *Fas^lpr^* mice, alone, did not generate any intestinal polyps. For most time points, *Apc^Min/+^*mice had slightly larger adenomas, reaching significance at 16 weeks (p<0.05) (Fig. 1B). At 30 weeks however, adenomas from *Apc^Min/+^/Fas^lpr^* mice were significantly larger than adenomas from *Apc^Min/+^* mice (p<0.01). Unlike *Apc^Min/+^* mice, where the location of adenomas is almost exclusively in the small intestine, *Apc^Min/+^/Fas^lpr^* mice after 16 weeks presented with large adenomas in the large intestine, additional to the ones located at the small intestine. Although there was significant tumor burden in *Apc^Min/+^* mice, it was much lower than in *Apc^Min/+^/Fas^lpr^* mice. Additionally, *Apc^Min/+^/Fas^lpr^* mice had a significantly reduced survival rate relative to *Apc^Min/+^* mice (Fig. 1C). The survival curves were compared using a log-rank test showing a significantly lower survival (p<0.001) in *Apc^Min/+^/Fas^lpr^* mice (*n* = 35) compared to *Apc^Min/+^*mice (*n = *89). The percent survival of *Apc^Min/+^/Fas^lpr^* mice strongly decreased between 25 and 30 weeks and reached 44% at 30 weeks. This is about 12 weeks earlier than that observed in *Fas^lpr^* mice [24]. Furthermore, unlike *Apc^Min/+^* mice, *Apc^Min/+^/Fas^lpr^* mice presented with invasive lesions in all animals at 30 weeks (Fig. 2). The invasive lesions are not to be confused with herniation, a common occurrence in *Apc^Min/+^* mice. All specimens were analyzed by a veterinary pathologist utilizing standardized guidelines [25]. The percentage of invasive lesions ranged from 3.0 to 15.4% (Table 1). However, metastasis to other organs was not detected.

**Table 1:** Percentage of invasive lesions in 30 week *Apc^Min/+^/Fas^lpr^* mice.

Mouse	Invasive lesions	Non-invasive lesions	Percentage of invasive lesions
AP266	1	32	3.00%
AP281	2	21	8.70%
AP285	2	26	7.10%
AP286	3	35	7.90%
AP287	4	22	15.40%

### Adenomas from *Apc^Min/+^/Fas^lpr^* Mice Show Increased Proliferation

**Figure 3:**
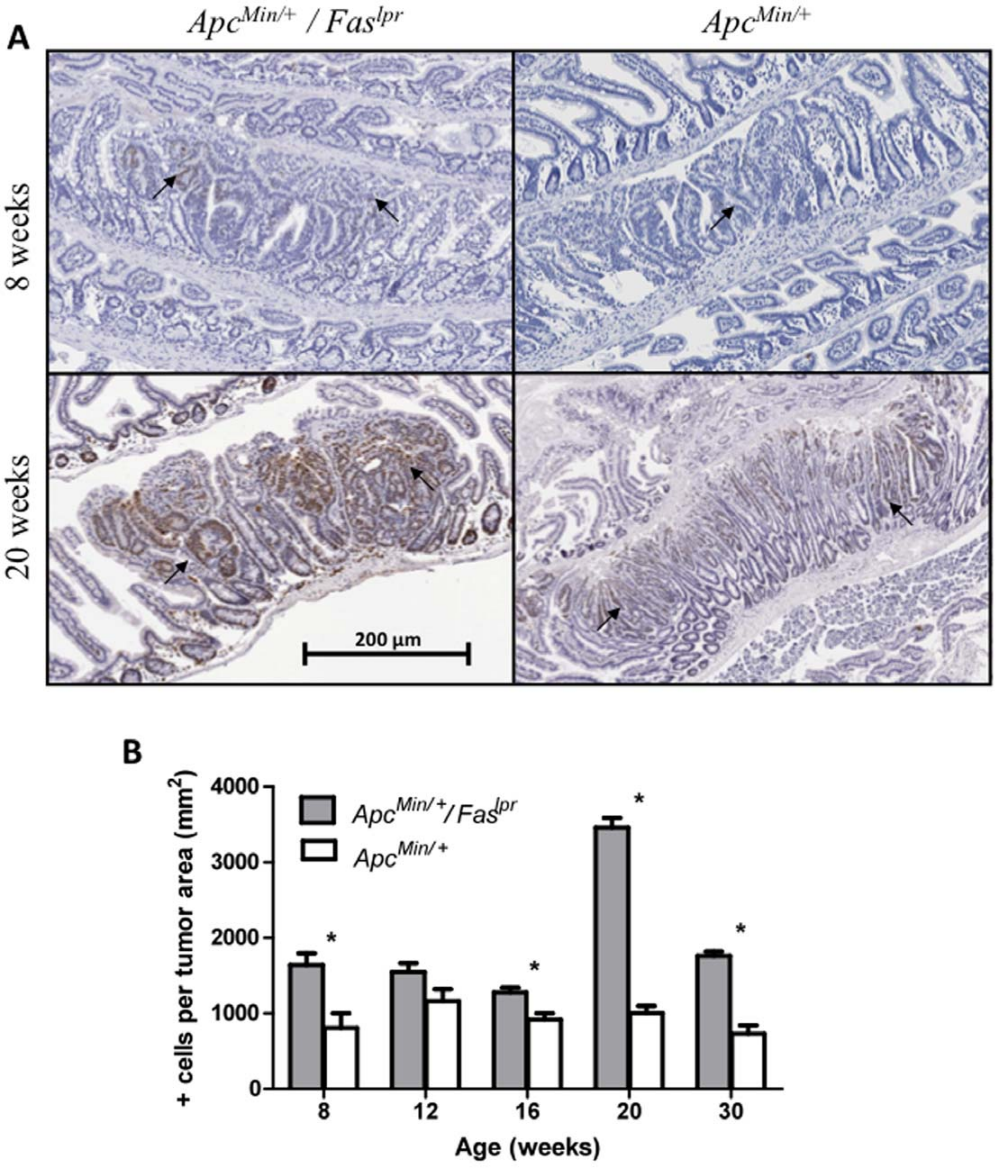
Immunohistochemistry for PCNA in adenomas from *Apc^Min/^* ^+^ and *Apc^Min/^* ^+^ */Fas^lpr^* mice. (**A**) PCNA immunostains comparing *Apc^Min/+^/Fas^lpr^* to *Apc^Min/+^* mice at 8 and 20 weeks. (**B**) Quantitation of PCNA positive cells per tumor area at five different time points. Error bars represent standard error of the mean.

**Figure 4:**
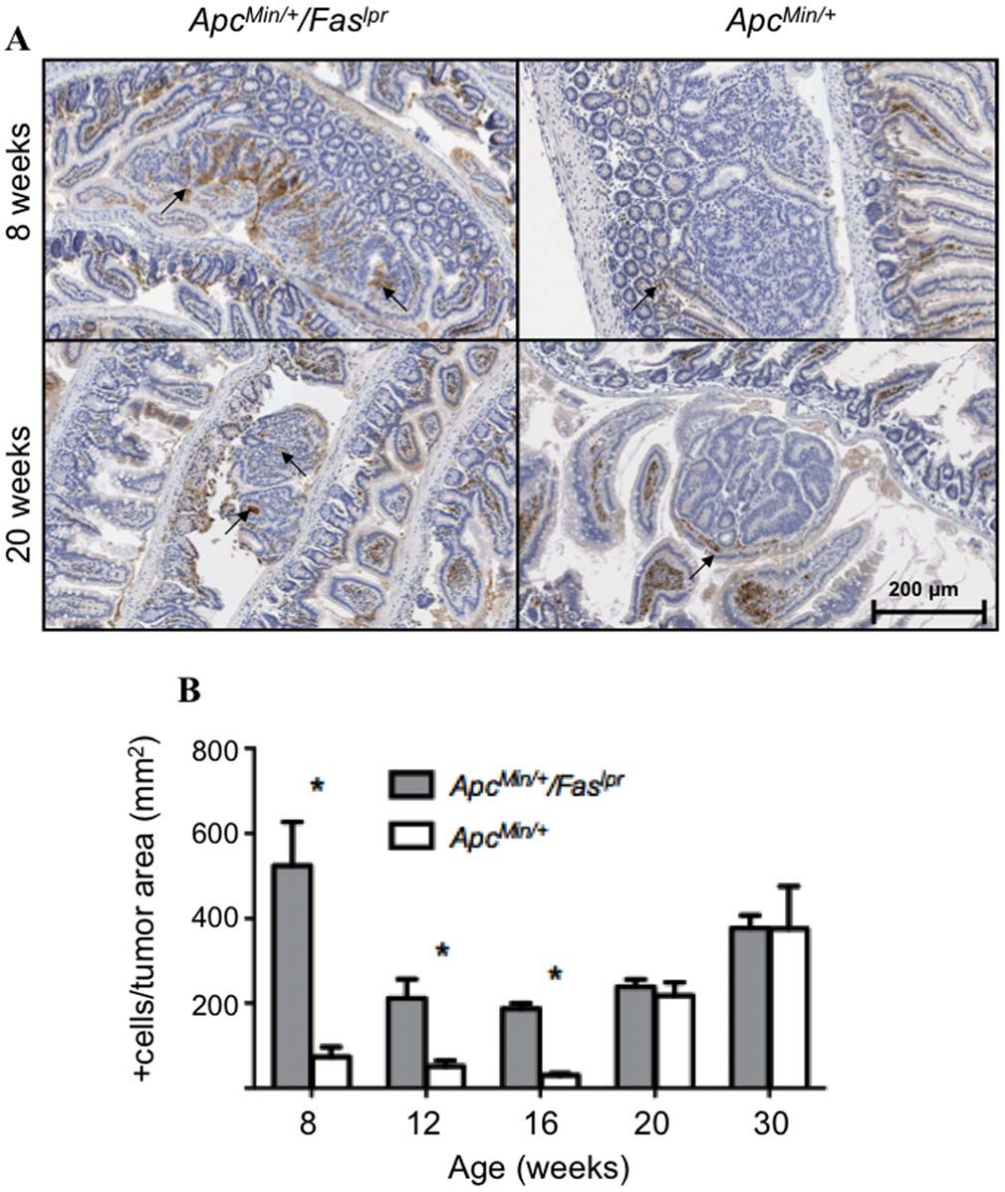
Immunohistochemistry for p53 in adenomas from *Apc^Min/^* ^+^ and ***Apc^Min/^*** ^+^ */Fas^lpr^* mice. (**A**) Immunostains for the apoptosis marker p53 in *Apc^Min/+^/Fas^lpr^* mice and *Apc^Min/+^* mice at 8 and 20 weeks. (**B**) Quantitation of p53 positive cells per tumor area at five different time points. Error bars represent standard error of the mean.

To determine if increased polyps in the *Apc^Min/+^/Fas^lpr^* mice were due to an increase in cellular proliferation and/or a decrease in apoptosis, cells positive for proliferating cell nuclear antigen (PCNA) (Fig. 3) and caspase-3 (data not shown) were determined by immunohistochemistry. Several (18–205) polyps from at least 4 mice were used for each time point and genotype. *Apc^Min/+^/Fas^lpr^* mice had significantly more PCNA+ cells per tumor area than *Apc^Min/+^* mice at most time points (p = 0.0014 at 8 weeks, p = 0.0038 at 16 weeks, p<0.0001 at 20 weeks and 30 weeks, n = 24–205 tumors for *Apc^Min/+^/Fas^lpr^* and 18–58 tumors for *Apc^Min/+^* in 4–5 mice per genotype) (Fig. 3). Apoptosis activity was determined by measuring cleaved caspase-3 levels. Surprisingly, *Apc^Min/+^/Fas^lpr^* did not show a significant change in caspase-3+ cells per tumor area at any time point, when compared to *Apc^Min/+^*mice. As another marker of apoptosis, p53 was also measured by immunohistochemistry. An increase in p53 levels was observed in *Apc^Min/+^/Fas^lpr^* mice compared to *Apc^Min/+^*mice (Fig. 4). The p53 increase was observed at early time points; 8 weeks (p = 0.0009), 12weeks (p = 0.012), and 16 weeks (p<0.0001) in *Apc^Min/+^/Fas^lpr^* mice compared to *Apc^Min/+^* mice (n = 26–177 tumors for *Apc^Min/+^/Fas^lpr^* and 18–78 tumors for *Apc^Min/+^* in 4–5 mice per genotype). After 20 weeks, both genotypes had similar levels of p53.

### Fas-L Is Decreased in *Apc^Min/+^* Mice Lacking Fas

**Figure 5:**
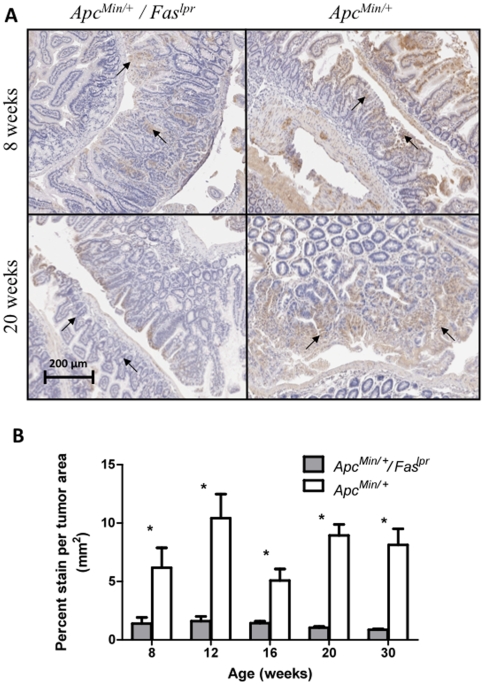
Immunohistochemistry for Fas-L in adenomas from *Apc^Min/^* ^+^ and ***Apc^Min/^*** ^+^ */Fas^lpr^* mice. (**A**) Immunostains for Fas-L comparing *Apc^Min/+^/Fas^lpr^* and *Apc^Min/+^* mice at 8 and 20 weeks. (**B**) Quantitation of Fas-L immunostains. Error bars represent standard error of the mean.

Another study has reported an increase in intestinal tumorigenesis once functional Fas-L is lost in *Apc^Min/+^* mice [17]. To determine if, in our Fas-deficient model, the levels of Fas-L within the adenomas were altered, immunostains of Fas-L were performed on intestinal sections of *Apc^Min/+^* and *Apc^Min/+^/Fas^lpr^* mice (Fig. 5). Adenomas from the *Apc^Min/+^/Fas^lpr^* mice had significantly less Fas-L per tumor area than *Apc^Min/+^*mice. This observation was consistent for all time points (p = 0.039 at 8 weeks, p<0.0001 at 12, 16, 20 and 30 weeks, n = 28–198 tumors for *Apc^Min/+^/Fas^lpr^* and 17–93 tumors for *Apc^Min/+^* in 4–5 mice per genotype).

### Akt, Foxo3a, and Inflammatory Markers in Intestinal Tumors from *Apc^Min/+^/Fas^lpr^* and *Apc^Min/+^* Mice

**Figure 6:**
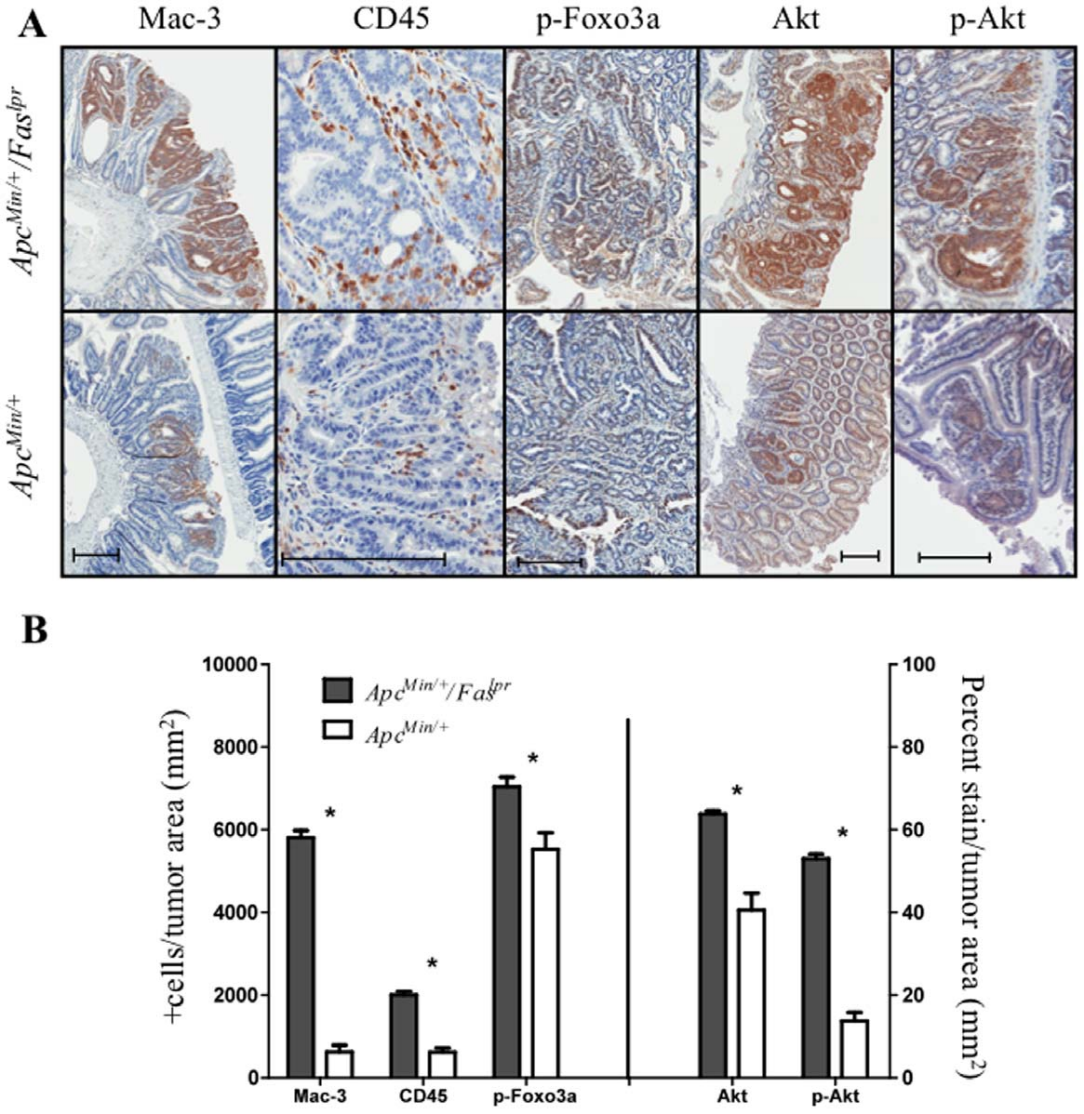
Inflammation in adenomas of *Apc^Min/^* ^+^ and *Apc* ^Min/+^ */Fas^lpr^* mice. (**A**) Immunostains for Mac-3, CD45, p-Foxo3a, Akt and pAkt in adenomas from *Apc^Min/+^* and *Apc^Min/+^/Fas^lpr^* mice at 30 weeks. (**B**) Quantitation of Mac-3, CD45, p-Foxo3a, Akt, and pAkt immunostains. Error bars represent standard error of the mean.

Fas-L, aside from triggering Fas dependent apoptosis, is considered to be an inducer of inflammation. To analyze inflammation within tumors, stains for CD45 and Mac-3 were performed on the intestines of *Apc^Min/+^/Fas^lpr^* and *Apc^Min/+^* mice (Fig. 6A). At 30 weeks, there were fewer positive cells for CD45 in tumors of *Apc^Min/+^* mice (617±99 +cells/mm^2^, n = 17 in 4 mice) than in *Apc^Min/+^/Fas^lpr^* mice (2006±86 +cells/mm^2^, n = 133 in 5 mice) (p<0.0001) (Fig. 6B). Mac-3, an antigen present on macrophages, had the same trend with lower levels in tumors of *Apc^Min/+^* mice (623±166 +cells/mm^2^, n = 21 in 4 mice) than in *Apc^Min/+^/Fas^lpr^* mice (5804±175 +cells/mm^2^, n = 151 in 5 mice) (p<0.0001).

In addition to PCNA, cell survival was further analyzed by staining for total Akt and its activated phosphorylated form (p-Akt) (Fig. 6A). The stains revealed a predictable trend in which tumors from *Apc^Min/+^/Fas^lpr^* mice showed higher levels of Akt (63.8%±7.6 n = 199 in 5 mice, versus 40.6%±4.1, n = 21 in 4 mice) and pAkt (53.0%±10.8, n = 186 in 5 mice, versus 13.7%±2.0, n = 28 in 5 mice) than *Apc^Min/+^* mice (p<0.0001) (Fig. 6B). Akt is reported to decrease expression of Fas-L by phosphorylating and, therefore, inhibiting Foxo3a, a member of the forkhead family of transcription factors responsible for Fas-L expression [26], [27]. In accordance with those reports, levels of phosphorylated Foxo3a (p-Foxo3a) were higher in the tumors of *Apc^Min/+^/Fas^lpr^* mice (7038±230 +cells/mm^2^, n = 139 in 4 mice) compared to the tumors in *Apc^Min/+^* mice (5523±398 +cells/mm^2^, n = 20 in 3 mice) (p = 0.017) (Fig. 6A,B).

### Hematology Profile

Prior to perfusion, blood was collected from all animals for hematological profiling. Measurements of leukocyte (neutrophils, lymphocytes, and monocytes) levels revealed that the averages remained within the normal ranges (data not shown).

*Apc^Min/+^/Fas^lpr^* mice, at most time points, were anemic as determined by red blood cell counts, hemoglobin, and percent hematocrit. However, *Apc^Min/+^* mice, as well as *Fas^lpr^* mice were also anemic. Platelets, in all cases, were within normal ranges (data not shown).

## Discussion

This study demonstrated that *Apc^Min/+^* mice develop more intestinal adenomas when Fas is absent. An increase in proliferation (measured by PCNA) was evident, especially at later time points where an increase in Akt and pAkt was also observed. Additionally, there was a decrease in Fas-L in *Apc^Min/+^/Fas^lpr^* mice. A significant increase in inflammation and Mac-3 was also observed in tumors of *Apc^Min/+^/Fas^lpr^* mice.

In the present study it was observed that at all time points (8, 12, 16, 20 and 30 weeks), *Apc^Min/+^* mice developed substantially more intestinal adenomas when Fas was eliminated. These adenomas also proved to be more aggressive. Incidences of intestinal prolapse were common in the *Apc^Min/+^/Fas^lpr^* mice, especially at the later time points. Overall, the poor health of these animals was evidenced by their diminished survival rate as they approached 30 weeks of age. These findings were not surprising and were in accord with expectations from disruption of the apoptotic pathway [28]. However, the levels of the apoptotic markers, activated caspase-3 and p53, were unexpected. There were no differences in activated caspase-3 levels, but an increase in p53 levels was observed in *Apc^Min/+^/Fas^lpr^* mice compared to *Apc^Min/+^* mice. Changes in cleaved caspase-3 were also absent in previous studies using *Apc^Min/+^* mice lacking Fas-L [17]. On the other hand, p53, also an apoptotic marker, was higher in tumors of *Apc^Min/+^/Fas^lpr^* mice compared to *Apc^Min/+^*mice at early time points. However, this difference was attenuated at 30 weeks, when Akt and pAkt were observed to be higher in *Apc^Min/+^/Fas^lpr^* mice compared to *Apc^Min/+^*mice. Akt is a protein kinase activated by a variety of growth factors [29], [30] that in turn triggers activation of several cancer-relevant downstream effector molecules, resulting in an environment that promotes proliferation and cell survival [31]. It has previously been reported that Akt is capable of downregulating p53 through phosphorylation of Mdm2 that results in a translocation to the nucleus [32], [33]. The increase of p53 might also be a compensatory mechanism for a lack of Fas pathway. The interdependence between Fas and p53 pathways has been demonstrated by others [34], [35].

**Figure 7:**
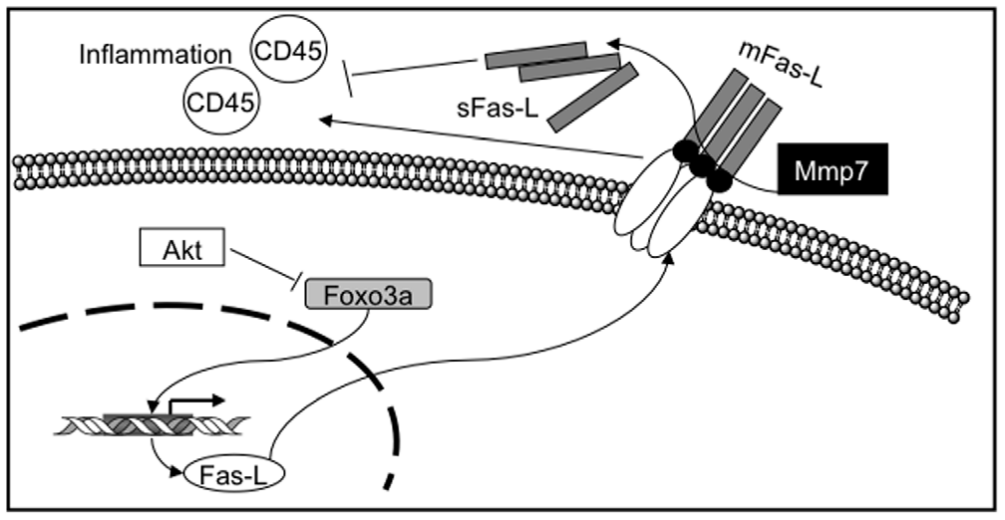
Proposed model for the increase in tumor burden in absence of Fas/CD95 in *Apc^Min/^* ^+^ mice. p-Akt inactivates Foxo3a by phosphorylation and p-Foxo3a is not able to translocate to the nucleus. Foxo3a is involved in the transcription of Fas-L. Fas-L forms a homotrimer transmembrane complex with a cleavage site (black circles), that is recognized by Mmp7. Mmp7 cleavage of mFas-L yields sFas-L (grey rectangles). The membrane and soluble forms of Fas-L have opposite effects on inflammation.

In accordance with reports indicating that Akt regulation in Fas-L expression is due to phosphorylation and, therefore, cytoplasmic retention of the forkhead transcription factor FKHRL1/Foxo3a [26], [27], intestinal adenomas of the *Apc^Min/+^/Fas^lpr^* mice at later time points presented with fewer Fas-L than tumors found in the *Apc^Min/+^*mice. This observation indicates that in the *Apc^Min/+^* mouse model, a Fas deficiency does not compromise tumor growth. On the contrary, by disrupting the Fas machinery, these tumors developed faster, and in greater numbers. These results are consistent with previous reports highlighting the anti-tumor effects of Fas-L [14], [36], as well as with recent findings of increased tumor burden in *Apc^Min/+^* mice deficient for Fas-L [17]. However, there are a number of studies that report opposite findings where Fas-L confers more rapid tumor formation in murine melanoma cells [37]. Higher Fas-L expression has also been reported in liver metastasis of colon cancer compared to the primary tumor [38]. Those findings might represent a compensatory mechanism for a disrupted Fas-mediated apoptotic pathway. Despite the general assumption that high levels of Fas-L would induce inflammation within the tumors, in the present study it was observed that *Apc^Min/+^/Fas^lpr^* mice, which had far lower levels of Fas-L than *Apc^Min/+^* mice, showed an increase in inflammation at 30 weeks of age. The mechanism behind the increased level of inflammation in tumors of *Apc^Min/+^/Fas^lpr^* mice is likely related to a balance between membrane Fas-L (mFas-L) and soluble Fas-L (sFas-L) (Fig. 7). mFas-L is a well known inducer of inflammation while sFas-L has the opposite effect [39]. Mmp7 is capable of cleaving mFas-L to yield sFas-L [40]. The *Apc^Min/+^* mouse model and colon cancer in general show elevated levels of matrilysin [41], [42]. Therefore, it is not unlikely to assume that in this context the balance between mFas-L and sFas-L would favor its soluble form and therefore a direct correlation between Fas-L and inflammation levels would be expected, as was observed in the present study. Fas-L expression is regulated by Foxo3a, a member of the forkhead family of transcription factors. Akt has been reported to decrease Fas-L expression by phosphorylation and therefore inactivation of Foxo3a. In accord with this mechanism, *Apc^Min/+^/Fas^lpr^* mice showed higher levels of Akt, p-Akt, and p-Foxo3a, and lower levels of Fas-L.

A relationship between inflammation and cancer has been identified in a number of studies, and it is widely accepted that inflammation creates conditions that promote tumor development (reviewed in [43]). Furthermore, Mac-3, an antigen present on macrophages, was present at higher levels in tumors of *Apc^Min/+^/Fas^lpr^* mice and their presence within tumors promoted tumor growth, invasion, and metastasis [44], [45].

The results of the present study correlate to those of Fingleton *et al.* [17] with differences that are worthy of further consideration. Fingleton *et al.* used 17 week *Apc^Min/+^* mice lacking Fas-L instead of its receptor, Fas. Both studies detected high levels of Fas-L in *Apc^Min/+^* mice. While deficiencies of either Fas or FasL resulted in an increase in tumor burden, in the current study, the increases in tumor number ranged from ∼300%, at early time points, to over 500%, at 16 weeks, whereas Fingleton *et al.* observed an increase nearly 100% at 17 weeks. These differences in tumor burden are most likely associated with difference in the inflammatory response observed in these studies. Fingleton *et al.* did not observe any significant changes in macrophage or lymphocyte infiltration, but did see a 3-fold decrease in neutrophils in *Apc^Min/+^* mice lacking Fas-L. In contrast, the current study demonstrated an obvious increase in the inflammatory response in *Apc^Min/+^/Fas^lpr^* mice. This supports the premise that the role played by in inflammation is more relevant in tumor development than it is in tumor evasion of the immune system. The current study, however, suggests that in intestinal adenomas, the balance between mFas-L and sFas-L levels, resulting from Mmp7 proteolysis, may regulate the pro- or anti-inflammatory properties of Fas-L.

Among the main objectives for generating the *Apc^Min/+^/Fas^lpr^* mice was to determine if a more aggressive variant of the *Apc^Min/+^* mouse model would evolve. At 30 weeks of age, all of the *Apc^Min/+^/Fas^lpr^* mice had invasive lesions, which was not observed in the *Apc^Min/+^* mouse model. It is likely that invasive lesions arise, at least in part, as a result of an increase in Akt and its active form, since this kinase has been shown to be involved in tumor invasion and metastasis [46], [47], [48].

In summary, this study demonstrated that an additional Fas deficiency in *Apc^Min/+^* mice causes a dramatic increase in the number of intestinal tumors. The increase in the incidence of adenoma development and the invasiveness of these adenomas, paralleling a decrease in Fas-L in these mice, does not support the Fas counterattack notion in this model. The increase in Mac-3 and CD45 suggests a tumor permissive environment caused by a Fas-L modulated inflammation.
